# Conundrums of Localized Surface Plasmon Resonance Biosensors

**DOI:** 10.1002/smll.202513520

**Published:** 2026-03-09

**Authors:** Nikhil Bhalla

**Affiliations:** ^1^ Nanotechnology and Integrated Bioengineering Centre (NIBEC), School of Engineering Ulster University 2‐24 York Street Belfast BT15 1AP United Kingdom

**Keywords:** biosensors, LSPR, nanotechnology, photonics, plasmonics

## Abstract

Localized surface plasmon resonance (LSPR) biosensing offers label‐free, real‐time detection of biomolecular interactions with high sensitivity and compact instrumentation. However, despite widespread use, several persistent and poorly understood phenomena limit the reproducibility, quantitation, and interpretability of LSPR signals. This perspective examines six recurring “conundrums” in LSPR biosensing, with a focus on their physical origins, experimental manifestations, and current approaches to mitigation. I review puzzling cases where analyte binding induces either red or blueshifts of the plasmon resonance; the difficulty of isolating the surface‐confined signal from bulk refractive‐index drift in microfluidics; the finite decay length of the plasmon field and the resulting saturation for thick or inhomogeneous layers; environmental cross‐sensitivities to temperature, pH, and ionic strength; run‐to‐run variability in nanostructure fabrication; and spectral congestion in multiplexed measurements. For each, I discuss the state of understanding, identify open questions, and outline strategies from advanced optical readouts to algorithmic baseline correction that could enable truly quantitative, drift‐immune, and reproducible LSPR biosensing.

## Graphical abstract

Localized surface plasmon resonance (LSPR) biosensing faces fundamental conundrums arising from finite field decay lengths, environmental cross‐sensitivities, and batch variability. Competing surface, bulk, and electrostatic effects can induce red or blue spectral shifts, complicating quantification, reproducibility, and interpretation in complex or multiplexed sensing environments. Created in BioRender. Bhalla, N. (2026) https://BioRender.com/p99uder.

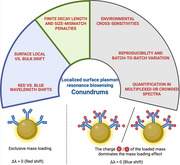

## 1 Introduction

**Figure 1:**
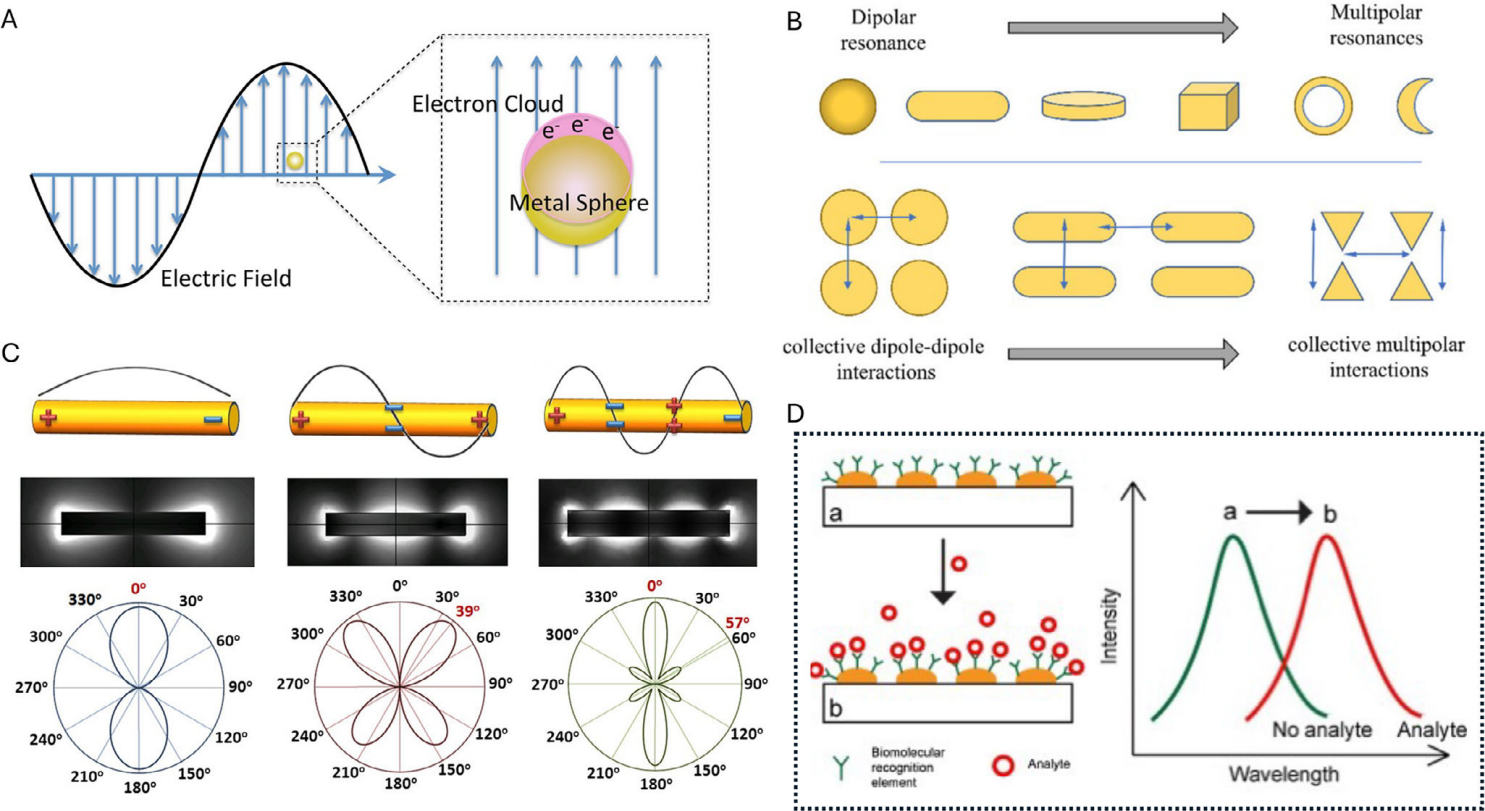
LSPR and biosensing (A) Illustration of the localized surface plasmon resonance of a metal nanoparticle in an oscillating electric field. Reproduced under the terms of the CC BY‐SA 4.0 license [5]. Copyright 2015, Wikidepia. (B) Schematic diagram for several NP nanostructures that are used in LSPR sensors, shifting from dipolar resonances to multipolar ones. These are presented in increasing order of tip sharpness. The lower section represents the interparticle interactions in array nanostructures, that will also cause additional changes on the spectral characteristics. Reproduced under the terms of the CC‐BY license [6]. Copyright 2021, MDPI (C) Schematic charge distribution, electric near‐field amplitude distribution, and far‐field scattering radiation pattern of a gold nanorod upon excitations of (a) its dipole mode (2060 nm), (b) quadrupole mode (1030 nm), and (c) sextupole mode (734 nm). Red numbers in the scattering patterns indicate the angles with maximal scattering power. Reproduced under the terms of the CC BY4.0 license [7]. Copyright 2014, Springer Nature. (D) Biomolecular recognition elements (e.g., antibody) on the surface of metal nanosubstrate (a) recognize and capture analyte (e.g., antigen) present in a liquid sample (b), producing a local increase in the refractive index at the metal surface. The increase of local refractive index induces a peak‐wavelength shift of the extinction spectra. Reproduced reproduced with permission CC‐BY‐NC‐ND [8]. Copyright 2018, Elsevier.

Localized surface plasmon resonance (LSPR) arises from the collective oscillation of conduction electrons in metallic nanostructures when driven by incident light at specific optical frequencies [1, 2], Figure 1A–C. This electron oscillation (resonance) leads to intense localized electromagnetic fields at the particle‐medium interface where in the spectral position of this resonance is exquisitely sensitive to the dielectric properties of the surrounding medium [3, 4]. In biosensing, this property is exploited by functionalising the metal surface with receptor molecules that selectively bind target analytes. Binding events alter the local refractive index within the plasmonic near field, thereby perturbing the resonance condition and causing a measurable wavelength shift, Δλ, in the scattering or extinction spectrum, Figure 1D. This shift is the fundamental transduction signal in LSPR biosensing.

In its simplest form, the change in resonance wavelength can be expressed as Equation (1)

(1) $$\Delta \lambda \approx S\;\Delta n_{\mathrm{eff}}$$

where S is the bulk refractive index sensitivity, typically reported in units of nm per refractive index unit (RIU), and $\Delta n_{\mathrm{eff}}$ represents the effective refractive index change sensed by the nanoparticle. The value of $\Delta n_{\mathrm{eff}}$ depends critically on the spatial overlap between the electromagnetic field and the refractive index perturbation induced by the bound analyte [9]. Since the electromagnetic field is strongly confined to the immediate vicinity of the particle, only a thin region, typically extending a few tens of nanometres from the surface, contributes significantly to the signal. The spatial decay of the local field, E(z), is often approximated by an exponential form

(2) $$E\left(z\right)\propto e^{-z/\ell_{d}}$$

where z is the distance from the particle surface and $\ell_{d}$ is the decay length, which depends on particle size, shape, and dielectric environment [4, 10]. This decay profile determines the weighting of the refractive index change as a function of distance. The effective refractive index change can therefore be written as

(3) $$\Delta n_{\mathrm{eff}}=\frac{\int_{0}^{\infty }\Delta n\left(z\right)e^{-z/\ell_{d}}\;dz}{\int_{0}^{\infty }e^{-z/\ell_{d}}\;dz}$$

For a homogeneous adsorbed layer of thickness d and refractive index contrast Δn, Equation (3) simplifies to

(4) $$\Delta n_{\mathrm{eff}}=\Delta n\left[1-e^{-d/\ell_{d}}\right]$$

which, when substituted into Equation (1), yields the widely used exponential saturation model

(5) $$\Delta \lambda \left(d\right)=S\;\Delta n\left[1-e^{-d/\ell_{d}}\right]$$

This model predicts a monotonic redshift that approaches a limiting value as the layer thickness greatly exceeds the decay length.

The physical picture outlined by Equations (1)–(5) has underpinned much of the early development of LSPR biosensing [2, 3]. The approach is attractive because of its conceptual simplicity, quantitative predictability, and direct link to electromagnetic theory. Over the past two decades, this framework has enabled the design of nanoparticle geometries and assay formats optimized for specific detection targets. Advances in nanofabrication, such as electron‐beam lithography, nanosphere lithography, and template‐assisted assembly, have made it possible to produce reproducible arrays of nanoparticles with controlled size, shape, and spacing [11]. The integration of LSPR sensors with microfluidics, portable spectrometers, and automated sample handling has further extended their application to real‐time, label‐free detection in fields ranging from clinical diagnostics to environmental monitoring [10].

However, as the field has matured, it has become increasingly clear that real experimental systems often deviate substantially from the idealised behavior predicted by the basic bulk‐refractive‐index model. Assays that, under the assumptions of Equations (1)–(5), should produce straightforward redshifts sometimes exhibit pronounced blueshifts [3]. Baseline drifts caused by bulk refractive index fluctuations in microfluidic delivery systems can obscure the small surface‐local signals of interest. Large biomolecular complexes or cells, whose dimensions exceed the decay length by an order of magnitude, can produce weaker than expected signals due to size‐mismatch penalties. Changes in temperature, pH, or ionic strength can induce spectral shifts of the same magnitude as the binding signal, complicating quantitative interpretation [1, 4]. Even in the absence of environmental variation, the reproducibility of measurements is often limited by batch‐to‐batch variability in nanoparticle synthesis or lithographic fabrication [11]. Furthermore, as multiplexed sensing schemes have been developed, where many nanoparticle geometries are interrogated simultaneously, overlapping spectral features have made it difficult to extract quantitative shifts without introducing fitting artefacts. These challenges are not merely technical inconveniences but arise from the fundamental physics of plasmonic sensing, the complexity of real experimental environments, and limitations in fabrication. They appear across different measurement methods such as extinction, scattering, or phase‐sensitive detection, and with many nanoparticle designs, from simple spheres to complex anisotropic structures. Since these challenges remain unresolved despite extensive research, these persistent difficulties can be regarded as conundrums in LSPR biosensing.

From a translational perspective, these conundrums matter because they directly determine analytical performance in realistic matrices, where the biosensing problem is typically drift‐limited rather than sensitivity‐limited. Bulk refractive‐index mismatch, temperature/pH/ionic‐strength fluctuations, and spectral ambiguity can produce apparent shifts that are comparable to or larger than true binding responses, increasing baseline noise and systematic drift and thereby raising the effective limit of detection (LoD). The same effects introduce bias in inferred concentration or kinetics (reducing accuracy) and can drive false positives/negatives when bulk and surface contributions are inseparable. Finally, batch‐to‐batch nanostructure variability alters resonance position, linewidth, and field confinement, undermining robustness and inter‐laboratory reproducibility even when nominal assay chemistry is identical. Thus, translating LSPR from proof‐of‐concept demonstrations to deployable biosensing requires strategies that explicitly manage drift, ambiguity, and variability so that intrinsic optical sensitivity can be converted into reliable LoD, accuracy, and robustness.

**Figure 2:**
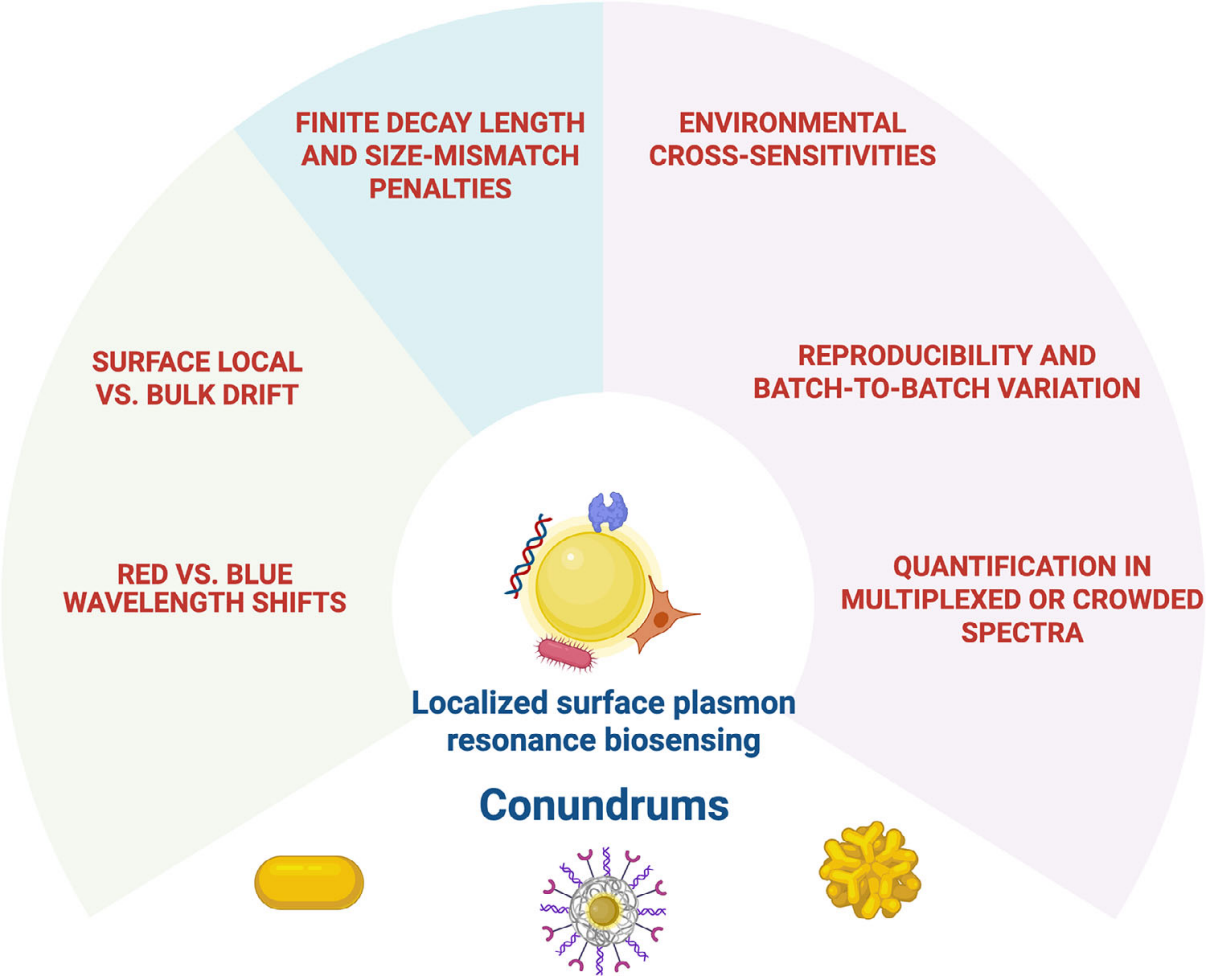
Key challenges in localized surface plasmon resonance (LSPR) biosensing. The schematic summarizes major conundrums encountered in LSPR‐based detection, including finite decay length and size‐mismatch penalties, environmental cross‐sensitivities, reproducibility and batch‐to‐batch variation, quantification in multiplexed or crowded spectra, red vs. blue wavelength shifts, and surface‐local vs. bulk drift effects.

In this work, I focus on six such conundrums that continue to challenge practitioners of LSPR sensing, Figure 2. Each conundrum is discussed in a dedicated section, where I outline the basic physical principles involved, illustrate the problem with representative examples, express it in mathematical terms, and examine current mitigation strategies. The goal is not only to document these difficulties but also to clarify their inter relationships and to point toward research directions that could lead to their resolution. By addressing these challenges in a systematic and quantitative manner, the aim is to contribute to the development of LSPR biosensing into a more robust, reproducible, and truly quantitative analytical platform. For quick quantitative context, Table 1 summarises typical order‐of‐magnitude, quantitative anchors encourtered in LSPR biosensing and their implications for LoD, accuracy and robustness.

**Table 1:** Order‐of‐magnitude quantitative anchors commonly encountered in LSPR biosensing. Values are indicative and depend on geometry, surface chemistry, and readout modality.

Phenomenon	Typical observable	Practical implication
Specific binding response	Peak‐shift signals often in the sub‐nm to few‐nm range (assay‐ and target‐dependent)	Sets the scale that drift and noise must be suppressed below for reliable limit of detection and quantitative accuracy
Baseline drift / bulk mismatch	Apparent shifts can be comparable to specific binding if temperature, buffer composition, or ionic strength vary during injections or long assays	Drift‐limited effective limit of detection; motivates multiparametric correction and rigorous referencing
Effective decay length $\ell_{d}$	Strongly geometry‐ and mode‐dependent (shorter for tightly confined hot spots; longer for extended modes)	Governs size‐mismatch penalties and the weighting of thick or inhomogeneous layers
Batch variability	Distributions in resonance wavelength ($\lambda_{0}$) and linewidth alter sensitivity and peak‐picking stability	Necessitates batch calibration, statistics over multiple sensing sites, and standardised reporting

## 2 Red‐ vs. Blue‐Shifting Assays

**Figure 3:**
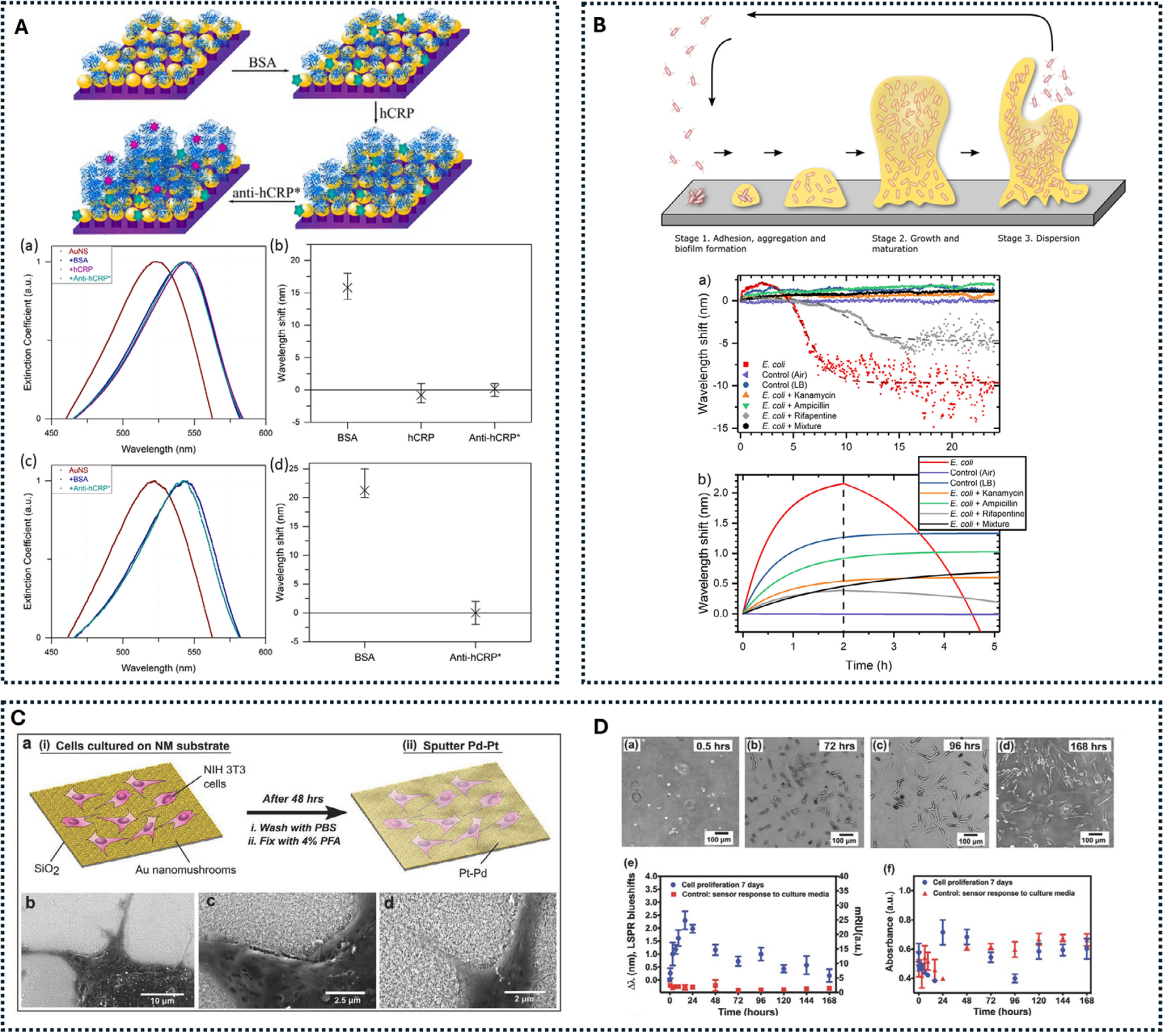
Red vs. blue wavelength shifts in LSPR: (A) illustrates a system in which the binding of small protein‐based biomolecules to the nanostructures induces both red and blue spectral shifts. Reproduced under the terms of the CC‐BY‐NC‐ND license [17]. Copyright 2019, ACS (B) shows the occurrence of blueshifts associated with biofilm formation. Parts are reproduced under the terms of the CC‐BY‐NC‐ND license [18]. Copyright 2018, ACS and CC‐BY‐NC‐ND license [19]. Copyright 2022, ACS. (C) and (D) together demonstrate cell attachment (C) on the surface–initially producing redshifts, followed by blueshifts as the cells proliferate (D) Reproduced under the terms of the CC‐BY license [16]. Copyright 2018, Wiley.

**Figure 4:**
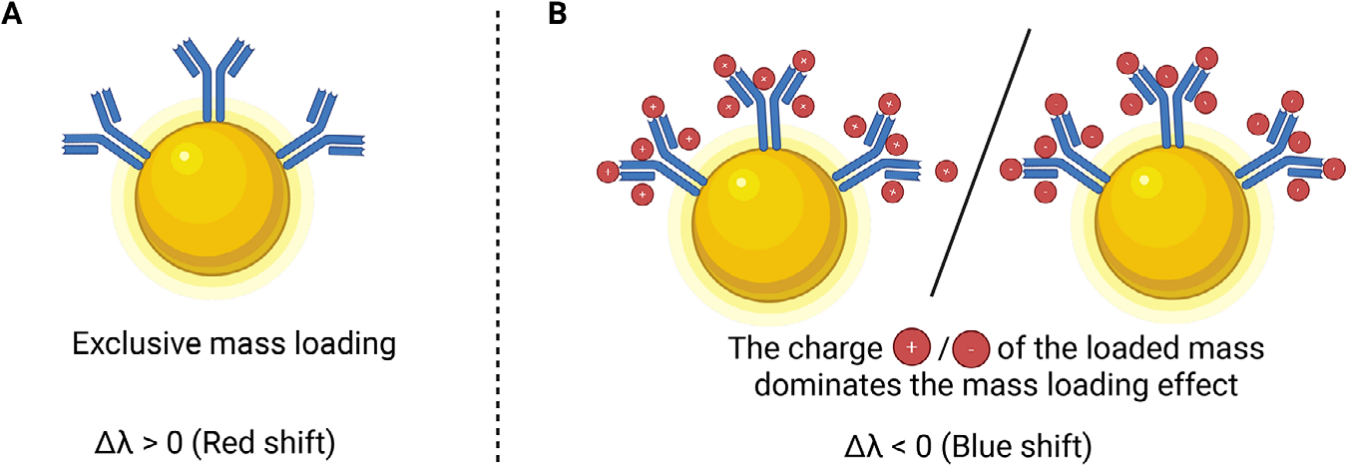
Charge‐dominated vs. mass‐dominated contributions to LSPR spectral shifts. (A) In the absence of significant charge transfer, biomolecular binding contributes primarily through mass loading and dielectric screening, leading to a redshift of the localized surface plasmon resonance (Δλ>0). (B) When charge effects associated with the loaded mass dominate—such as net positive or negative charge accumulation at the metal surface—the increase in free‐carrier density and/or reduction in effective screening can outweigh dielectric loading, resulting in a blueshift of the LSPR (Δλ<0). Schematic charges indicate the sign of the dominant interfacial charge contribution rather than absolute stoichiometry. Created in BioRender. Bhalla, N. (2026) https://BioRender.com/e84n9qx.

In the conventional description of LSPR sensing, adsorption of analytes with refractive index $n_{\mathrm{layer}}$ higher than that of the surrounding medium $n_{m}$ produces a redshift of the resonance wavelength, reflecting an increase in the effective dielectric function. This behavior can be expressed as

(6) $$\Delta \lambda \propto \Delta n_{\mathrm{eff}}=\int_{0}^{\infty }\Delta n\left(z\right)W\left(z\right)\;dz$$

where W(z) is the near‐field weighting function decaying away from the nanoparticle surface. For a uniform adsorbed layer, W(z) is well approximated by an exponential form with characteristic decay length $\ell_{d}$, yielding a strictly positive Δλ when $\Delta n=n_{\mathrm{layer}}-n_{m}>0$. However, several recent studies, including my own works, have shown conditions under which binding induces a counterintuitive blueshift (Δλ<0), see Figure 3A–D. Kosame and colleagues observed such a shift in silver nanoparticles dispersed in indigo carmine solutions, explained by a nonlinear Kerr‐type effect in which the refractive index of the medium decreases with increasing optical intensity [12]. This can be modeled as

(7) $$n\left(I\right)=n_{0}+n_{2}I$$

where $n_{2}<0$ gives an intensity‐dependent decrease in refractive index, thereby producing $\Delta n_{\mathrm{eff}}<0$ and a net blueshift. A second mechanism arises from plasmonic mode coupling in anisotropic nanostructures. When two dipolar plasmons interact, their hybridised modes split as

(8) $$\omega_{\pm }^{2}\approx \omega_{0}^{2}\left(1\pm \frac{\alpha }{R^{3}}\right)$$

where $\omega_{0}$ is the single‐particle resonance, α the polarizability, and R the interparticle spacing. The antibonding (+) mode corresponds to a higher frequency (blueshift), while the bonding (−) mode produces a redshift. This effect explains recent reports of shell‐induced blueshifts when a single atomic silver shell is grown on gold nanorods by antigalvanic processes [13]. Charge‐transfer dynamics also play a crucial role. Sakamoto et al. demonstrated that LSPR excitation in CuS nanocrystals can trigger a cooperative Jahn–Teller distortion and a crystal‐structure change. This structural/ electronic reconfiguration is accompanied by a measurable modification of the plasmonic response, highlighting that lattice distortions can reshape plasmon spectra beyond simple refractive‐index effects [14]. Similarly, Ostovar et al. showed that plasmon‐induced direct electron injection into acceptor states modifies the damping constant γ in the Drude dielectric function,

(9) $$\varepsilon \left(\omega \right)=\varepsilon_{\infty }-\frac{\omega_{p}^{2}}{\omega^{2}+i\gamma \omega }$$

leading to broadening of the homogeneous plasmon linewidth which may further lead to shifts of the resonance toward shorter wavelengths [15]. My own work has further highlighted how whole‐cell interactions can induce pronounced blueshifts attributable to charge‐wave coupling at the cell‐nanoparticle interface, highlighting the contribution of electrostatics alongside refractive index changes [16]. Collectively, these findings demonstrate that LSPR peak shifts cannot be attributed solely to local refractive index increases. Instead, the sign of Δλ reflects a superposition of dielectric loading, nonlinear optical responses (Equation (7)), plasmon hybridization (Equation (8)), and interfacial charge‐transfer dynamics (Equation (9)). The key implication is that blueshifts are not artefacts but physically meaningful signatures of additional processes that must be considered in biosensing interpretation. A comprehensive predictive model for LSPR sensing therefore requires the integration of electromagnetic coupling, nonlinear optics, and interfacial electron dynamics, validated under controlled assay conditions. Accordingly, as per above discussion, while conventional LSPR sensing is often interpreted as dielectric mass loading (typically yielding Δλ>0), interfacial charge accumulation or charge‐transfer processes can dominate the optical response and drive blueshifts (Δλ<0), see more details in Figure 4 and Table 2.

**Table 2:** Charge‐dominated mechanisms leading to blueshifts in LSPR systems.

Effect / Mechanism	Physical origin of charge	How it modifies LSPR	Why it may lead to blueshift
**Kerr effect (optical Kerr** / $\chi^{\left(3\right)}$ **nonlinearity)**	Strong optical fields induce an intensity‐dependent change in the local refractive index *via* nonlinear polarization	Alters the effective dielectric environment experienced by the plasmon	Reduced effective refractive index at high field intensities lowers the plasmon resonance wavelength, producing a blueshift
**Antigalvanic coupling**	Electron donation from adsorbates (e.g. ligands, molecules, ions) into metal nanoparticles, increasing electron density	Raises free‐carrier concentration in the metal	Increased plasma frequency ($\omega_{p}\propto \sqrt{n}$) shifts the LSPR to shorter wavelengths
**Electrostatic charging (field‐effect / capacitive gating)**	External bias or local charge accumulation at the metal–electrolyte interface	Modulates surface charge density and Fermi level	Higher electron density stiffens the plasmon oscillation, leading to a blueshifted resonance
**Hot‐electron accumulation (non‐equilibrium carriers)**	Photoexcited electrons transiently populate higher‐energy states	Temporarily alters carrier distribution and electronic screening	Enhanced electron population near the Fermi level increases $\omega_{p}$, causing a transient blueshift
**Charge transfer from adsorbates (chemical interface damping regime)**	Partial electron transfer between metal and adsorbed species	Modifies surface electron density and interfacial screening	Net electron donation dominates over dielectric loading, shifting the LSPR to shorter wavelengths
**Ion adsorption / electrical double‐layer formation**	Preferential adsorption of cations near the nanoparticle surface	Creates strong interfacial electric fields	Field‐induced carrier accumulation outweighs refractive‐index increase, producing a blueshift
**Reduced dielectric screening (hydration or depletion layers)**	Local depletion of high‐index solvent or reorientation of dipoles	Lowers effective surrounding permittivity	Reduced screening increases restoring force of plasmon oscillation, shifting resonance blue
**Quantum confinement / ultrasmall particle effects**	Discrete electronic states and reduced screening at small sizes	Alters collective electron response	Increased effective plasmon stiffness shifts resonance to shorter wavelengths

## 3 Surface‐Local Signal vs. Bulk Drift

**Figure 5:**
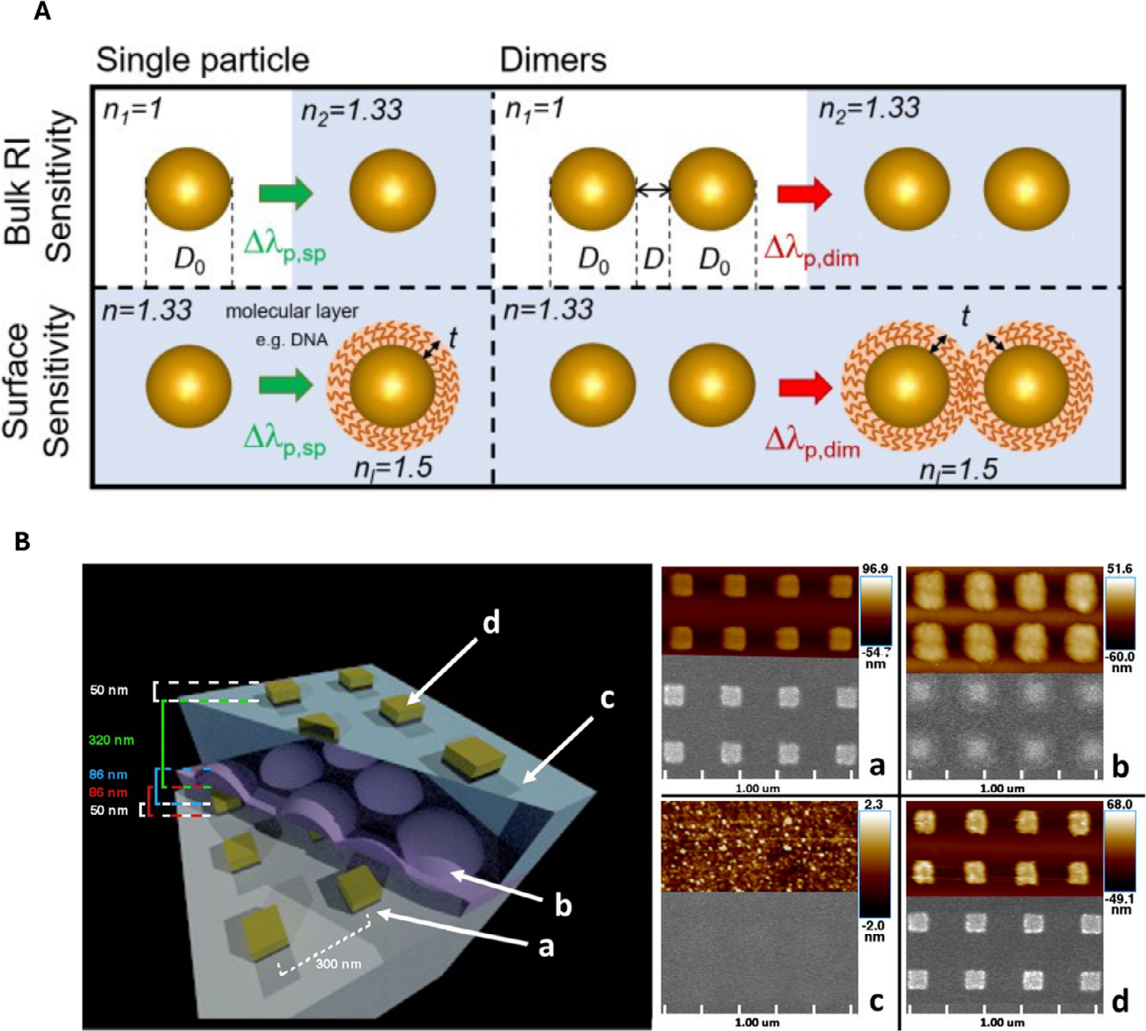
Bulk and surface sensitivity in LSPR sensor architectures. (A) Schematic illustration of the simulation setup and definitions of the bulk refractive index sensitivity (RIS) and surface sensitivity. Parameters: $D_{0}$‐nanosphere diameter; D‐interparticle distance (gap); $\Delta \lambda_{\text{res}}$‐extinction peak shift for single particles (sp) and dimers (dim); t‐dielectric layer thickness; $n_{l}$‐refractive index of the dielectric layer. Reproduced under the terms of the CC‐BY license [20]. Copyright 2021, MDPI (B) Structure and characterization of a multilayered LSPR sensing device. *Left*: cross‐sectional schematic showing (a) the gold nanostructure LSPR reference layer encapsulated by (b) a conformal silicon nitride film, followed by (c) an HSQ planarization layer and (d) the gold nanostructure LSPR sensing layer. Each nanostructure measures 100×100×50 nm (L × W × H). *Right*: AFM (top) and SEM (bottom) micrographs of the device layers (a‐d) taken after deposition. The AFM images confirm that the HSQ layer effectively planarizes the underlying nitride surface. Reproduced under the terms of the CC‐BY license [26]. Copyright 2018, ACS.

One of the most persistent challenges in quantitative LSPR biosensing is the separation of the true surface‐local refractive index signal from baseline drift caused by bulk refractive index (RI) fluctuations in the surrounding medium, see Figure 5A which shows bulk and surface sensitivity setups used for simulation in literature [20]. The fundamental appeal of LSPR lies in its surface confinement: the near field decays to negligible amplitude within a distance $\ell_{d}$ of the metal surface, and therefore the sensor is expected to be primarily responsive to changes within this region. This principle underpins monolayer detection, but in practice, measurements integrate contributions from both the confined near field and residual interactions with propagating modes or substrate‐mediated fields [21, 22, 23]. As a result, even subtle bulk RI variations for example those induced by fluid exchange, buffer mismatch, or temperature driven density changes can produce apparent spectral shifts comparable to those arising from molecular binding [24, 25].

A useful formalization expresses the measured resonance, $\lambda_{\mathrm{res}}\left(t\right)$, as a sum of surface, bulk, and instrumental contributions:

(10) $$\lambda_{\mathrm{res}}\left(t\right)=\lambda_{0}+\Delta \lambda_{\mathrm{surf}}\left(t\right)+\Delta \lambda_{\mathrm{bulk}}\left(t\right)+\eta \left(t\right)$$

where $\lambda_{0}$ is the baseline, $\Delta \lambda_{\mathrm{surf}}\left(t\right)$ is the surface‐local shift, $\Delta \lambda_{\mathrm{bulk}}\left(t\right)$ the bulk shift, and η(t) represents noise.

The surface contribution follows

(11) $$\Delta \lambda_{\mathrm{surf}}\left(t\right)=S_{\mathrm{surf}}\;\Delta n_{\mathrm{surf}}\left(t\right)\left[1-e^{-d\left(t\right)/\ell_{d}}\right]$$

while the bulk term can be approximated by

(12) $$\Delta \lambda_{\mathrm{bulk}}\left(t\right)\approx S_{\mathrm{bulk}}\;\Delta n_{\mathrm{bulk}}\left(t\right)$$

Because $\Delta n_{\mathrm{bulk}}$ values as small as $10^{-4}$ RIU can yield 0.1–1 nm shifts, reference strategies are indispensable. Dual‐channel referencing remains the most widely implemented approach:

(13) $$\Delta \lambda_{\mathrm{diff}}\left(t\right)=\Delta \lambda_{\mathrm{surf}}\left(t\right)$$

where subtraction cancels common‐mode drift. However, imperfect channel symmetry or fouling can leave residual artefacts. Alternative referencing architectures include embedding inert nanoparticles alongside active ones to provide in‐flow correction:

(14) $$\Delta \lambda_{\mathrm{tot}}\left(t\right)=f_{\mathrm{act}}\left[\Delta \lambda_{\mathrm{surf}}\left(t\right)+\Delta \lambda_{\mathrm{bulk}}\left(t\right)\right]+f_{\mathrm{ref}}\Delta \lambda_{\mathrm{bulk}}\left(t\right)$$

from which $\Delta \lambda_{\mathrm{surf}}\left(t\right)$ can be isolated. Moreover, here $f_{\mathrm{act}}$ and $f_{\mathrm{ref}}$ denote the fractional optical contributions of the active and inert nanoparticle populations to the ensemble plasmonic signal, weighting their respective surface‐sensitive and bulk‐only responses. Furthermore, multimetric spectral analysis exploits

(15) $$m\left(t\right)=\left[\lambda_{\mathrm{peak}}\left(t\right),\;\Gamma \left(t\right),\;A\left(t\right)\right]$$

where bulk changes primarily shift $\lambda_{\mathrm{peak}}$, while binding can also alter linewidth Γ and amplitude A. Kinetic fitting can help separate overlapping processes. Binding is described by

(16) $$\frac{d\theta }{dt}=k_{\mathrm{on}}C\left(1-\theta \right)-k_{\mathrm{off}}\theta$$

with signal

(17) $$\Delta \lambda_{\mathrm{surf}}\left(t\right)=\Delta \lambda_{\max}\;\theta \left(t\right)$$

Despite these advances, a universally applicable calibration‐free solution remains elusive. In biological fluids, bulk and surface changes are strongly correlated. Essentially, injection of analyte solutions often introduces both RI mismatch and binding simultaneously. Environmental perturbations such as temperature or ionic fluctuations can mimic either contribution. Recent proposals suggest integrating thermal and conductivity sensors directly into LSPR chips [27, 28], or using hybrid plasmonic modes with distinct penetration depths to discriminate bulk from surface effects [29, 30]. Overall, the conundrum is that while multiple strategies‐referencing (for instance the self referencing nanostructure architecture in Figure 5B), dual‐mode engineering, multimetric analysis, and adaptive algorithms can each reduce drift, none yet provides a universal solution. Achieving reliable separation of $\Delta \lambda_{\mathrm{surf}}$ from $\Delta \lambda_{\mathrm{bulk}}$ will likely require combining structural design with environmental sensing and data‐driven modeling.

## 4 Finite Decay Length and Size‐Mismatch Penalties

**Figure 6:**
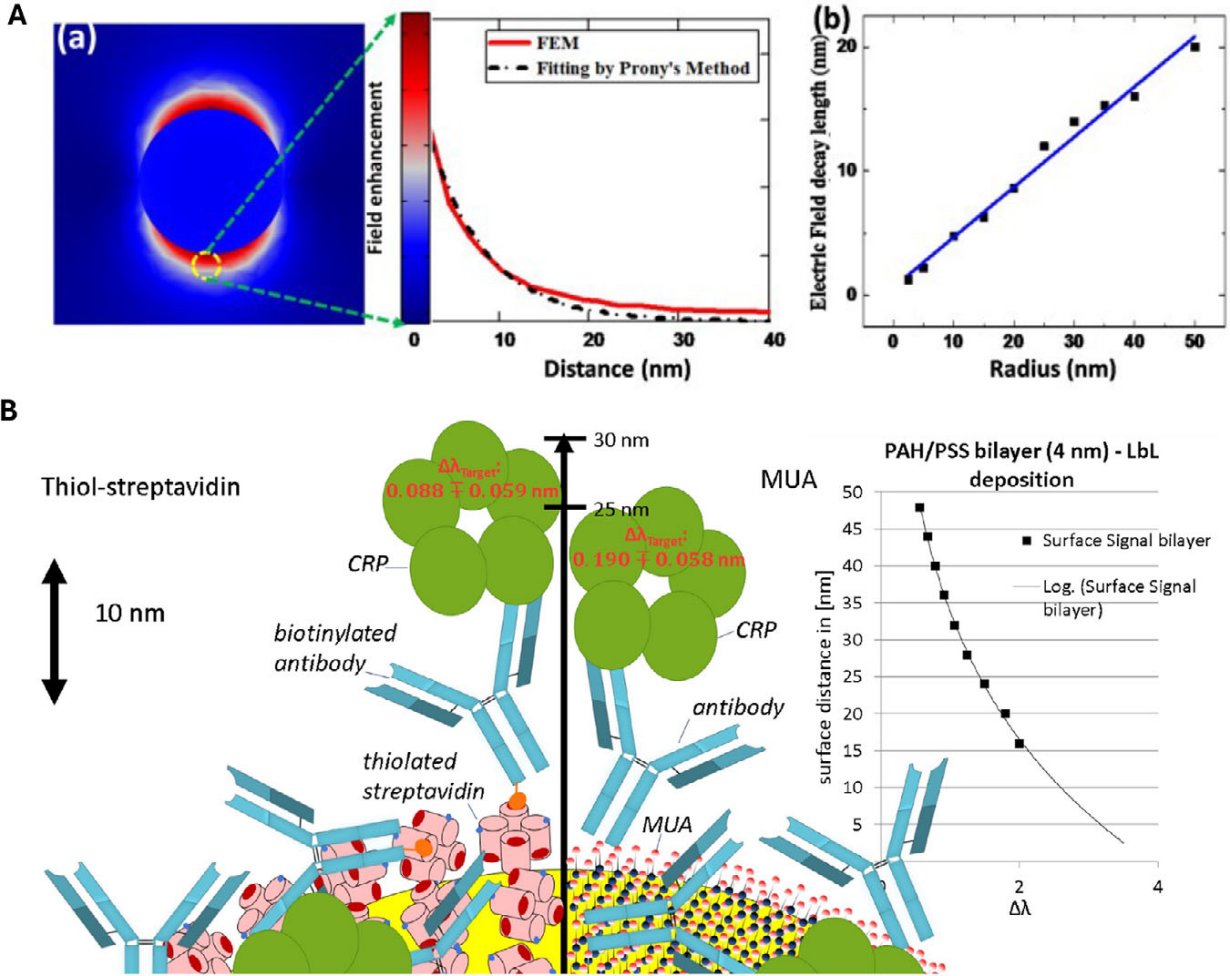
Decay length and size (A) (a) The calculation of $l_{d}$ values for a gold nanosphere (r=30nm) using a 3D full‐wave field analysis in a homogeneous medium (n=1.33) and (b) its relation between the particle radius. Reproduced under the terms of the CC‐BY license [35]. Copyright 2022, MDPI. (B) scheme of the studied immobilization approaches for anti‐CRP antibodies. Left: Biotinylated anti‐CRP antibodies are attached to the gold surface by thiolated streptavidin. Right: After a surface modification by a self‐assembled monolayer of MUA, EDC chemistry is utilized to attach unmodified anti‐CRP antibodies. The inset shows the decrease of the sensor signal with increasing surface distance for layer‐by‐layer (LbL) deposition with charged polyelectrolyte (PEL) bilayers. Reproduced under the terms of the CC‐BY license [36]. Copyright 2022, Springer Nature.

The surface selectivity of LSPR biosensing stems from the rapid decay of the near field away from the metal‐dielectric interface. As established in the introduction section, a homogeneous overlayer at distance z contributes to the signal with an exponential weight, which yields the familiar saturation of the wavelength shift with film thickness (Equations (4) and (5)). Rather than restating those expressions, here I focus on the consequences for large and inhomogeneous targets and on how the characteristic decay length $\ell_{d}$ varies with nanostructure geometry and readout. Recent experiments across microfluidic [31], colloidal [32], and engineered dimer/ metasurface platforms [33, 34] consistently show that the sampling depth is typically on the order of a few to a few‐tens of nanometres, albeit strongly geometry‐ and mode‐dependent, such that values below 10 nm and above 50 nm have both been reported, see Figure 6A which shows variation of decay length as a function of distance from the surface and also as function of LSPR active nanoparticle size (Au nanoparticle in this case).

A practical point is that $\ell_{d}$ is most usefully treated as an effective (observable‐dependent) decay length, because different readouts weight the near‐field differently (near‐field amplitude, near‐field intensity, or a far‐field spectral metric such as the LSPR peak shift). In the near field of a localized plasmon mode, the field magnitude typically decreases approximately exponentially with distance from the metal surface over the sensing volume, motivating operational definitions of a characteristic 1/e decay length used widely in LSPR sensing models [2, 4]. In simulations (FDTD/FEM/BEM), one can compute the complex electric field E(r) and analyze its magnitude along the surface‐normal coordinate z (with z=0 at the metal–dielectric interface). Denoting the field magnitude as |E(z)| and the intensity as ${\left|E\left(z\right)\right|}^{2}$, an operational local decay length can be defined from the log‐slope:

(18) $$\ell_{d}^{\left(E\right)}\left(z\right)\;=\;-{\left(\frac{d}{dz}\ln\left|E\left(z\right)\right|\right)}^{-1},\;\ell_{d}^{\left(I\right)}\left(z\right)\;=\;-{\left(\frac{d}{dz}\ln{\left|E\left(z\right)\right|}^{2}\right)}^{-1}$$

where ln is the natural logarithm and d/dz is the derivative along the surface normal. If $\left|E\left(z\right)\right|\propto \exp\left(-z/\ell_{d}\right)$ is strictly exponential, these expressions reduce to the constant $\ell_{d}$. In practice, $\ell_{d}^{\left(E\right)}\left(z\right)$ and $\ell_{d}^{\left(I\right)}\left(z\right)$ can be mildly z‐dependent and are therefore best interpreted as effective decay lengths within the relevant sensing region. Such near‐field extraction is straightforward in boundary‐element implementations (e.g., MNPBEM) and in standard time‐/frequency‐domain solvers [37].

Experimentally, $\ell_{d}$ can be extracted by inserting calibrated spacer thicknesses (e.g., layer‐by‐layer polyelectrolyte multilayers or conformal dielectric overlayers) between the receptor plane and a known index perturbation, and fitting the resulting distance response to the same exponential/ saturation formalism already used here (Equations 4 and 5). This distance‐series calibration yields an in situ $\ell_{d}$ under the exact buffer, temperature, and optical readout conditions used for sensing, and it provides a quantitative estimate of size‐mismatch penalties for large or inhomogeneous objects [38].

For finite objects, a convenient way to quantify the penalty from sampling only the portion of the analyte within the near field is to use a volumetric weighting. If $V_{\mathrm{eff}}$ denotes the fraction of an analyte's volume lying within $z<\ell_{d}$, then the effective index perturbation detected is

(19) $$\Delta n_{\mathrm{eff}}=\phi_{\mathrm{eff}}\;\Delta n\;\phi_{\mathrm{eff}}=\frac{V_{\mathrm{eff}}}{V_{\mathrm{tot}}}$$

For a spherical particle of radius a that contacts the sensor surface, the 1D convolution of the field weight with a constant refractive‐index contrast gives

(20) $$\Delta n_{\mathrm{eff}}\left(a\right)=\Delta n\left[1-\exp\;\left(-\frac{2a}{\ell_{d}}\right)\right]$$

highlighting that even very large objects ($a\gg \ell_{d}$) do not increase the response beyond the asymptote set by $\ell_{d}$. Experimentally, this saturation has been mapped by inserting calibrated spacer stacks (e.g., polyelectrolyte bilayers) between receptors and targets; the observed distance law follows the expected exponential with an extracted $\ell_{d}$ that depends on nanoparticle size and spectral mode [32]. Importantly, the marginal sensitivity to additional thickness decays exponentially; differentiating the saturation model yields

(21) $$\frac{d\Delta \lambda }{dd}=\frac{S\;\Delta n}{\ell_{d}}\exp\;\left(-\frac{d}{\ell_{d}}\right)$$

which quantifies why adding thicker cushions (e.g. linkers or passivation layers) rapidly diminishes returns, see Figure 6B which shows wavelength shifts with respect to biocomplex height or distance from the surface.

A second practical nuance is that $\ell_{d}$ is not universal for a given material but varies with geometry and resonance. Quantitatively, effective sensing depths $\ell_{d}$ reported for LSPR platforms commonly span from ∼5–10 nm for tightly confined hot‐spot/ nanogap‐type geometries to ∼20–40 nm (and sometimes higher) for less confined modes and larger structures, highlighting that geometry/ mode selection can change the weighting of thick or inhomogeneous layers by several‐fold. Coupled‐mode calculations and full‐field simulations show that elongated particles, redshifted longitudinal modes, and strongly coupled dimers/ nanogaps redistribute the near field and can extend the effective sampling depth along selected axes, partially mitigating size mismatch [33, 34]. Conversely, blueshifted or higher‐order modes can confine fields more tightly, increasing surface selectivity but penalising thick overlayers. In fluids, microenvironment changes that accompany flow exchange also modulate the apparent depth response and therefore must be accounted for during calibration [31].

Several calibration and design strategies have emerged to turn these constraints into quantitative tools. Distance‐series with layer‐by‐layer spacers provide an in‐situ estimate of $\ell_{d}$ under assay conditions [32]. Dual‐band or multi‐modal plasmonic “nanorulers” combine two independently tuned resonances to decouple film thickness from refractive index [39], reducing the ambiguity inherent to single‐peak readouts and improving quantification for large or heterogeneous adsorbates [40]. From a benchmarking perspective, incorporating $\ell_{d}$ explicitly into figures of merit (rather than relying only on bulk RI sensitivity) has been proposed so that devices with longer sampling depth are fairly compared to strongly confined sensors [41]. Theoretical treatments that separate bulk and local refractive‐index sensitivity likewise clarify why two sensors with identical bulk sensitivity can differ in response to the same nanoscale layer [42].

In practice, when targets exceed the near‐field sampling depth as for viruses, extracellular vesicles, or whole cells, the part of the object within $z\;≲\;\ell_{d}$ dominates the signal, and the remainder contributes little. This explains weak or sub‐linear scaling of Δλ with geometric size and motivates (i) pushing resonances to longer wavelengths or employing anisotropic/ coupled antennas to extend $\ell_{d}$ along the relevant direction [33, 34], (ii) using thin, high‐index capture layers and minimal spacers to keep binding sites within the sensitive zone [32], and (iii) adopting multi‐metric or multi‐modal readouts that can separate thickness from composition [40]. Without such measures, the “size‐mismatch penalty” remains a dominant source of underestimation, even when other sources of drift are well controlled [31].

## 5 Environmental Cross‐Sensitivities

**Figure 7:**
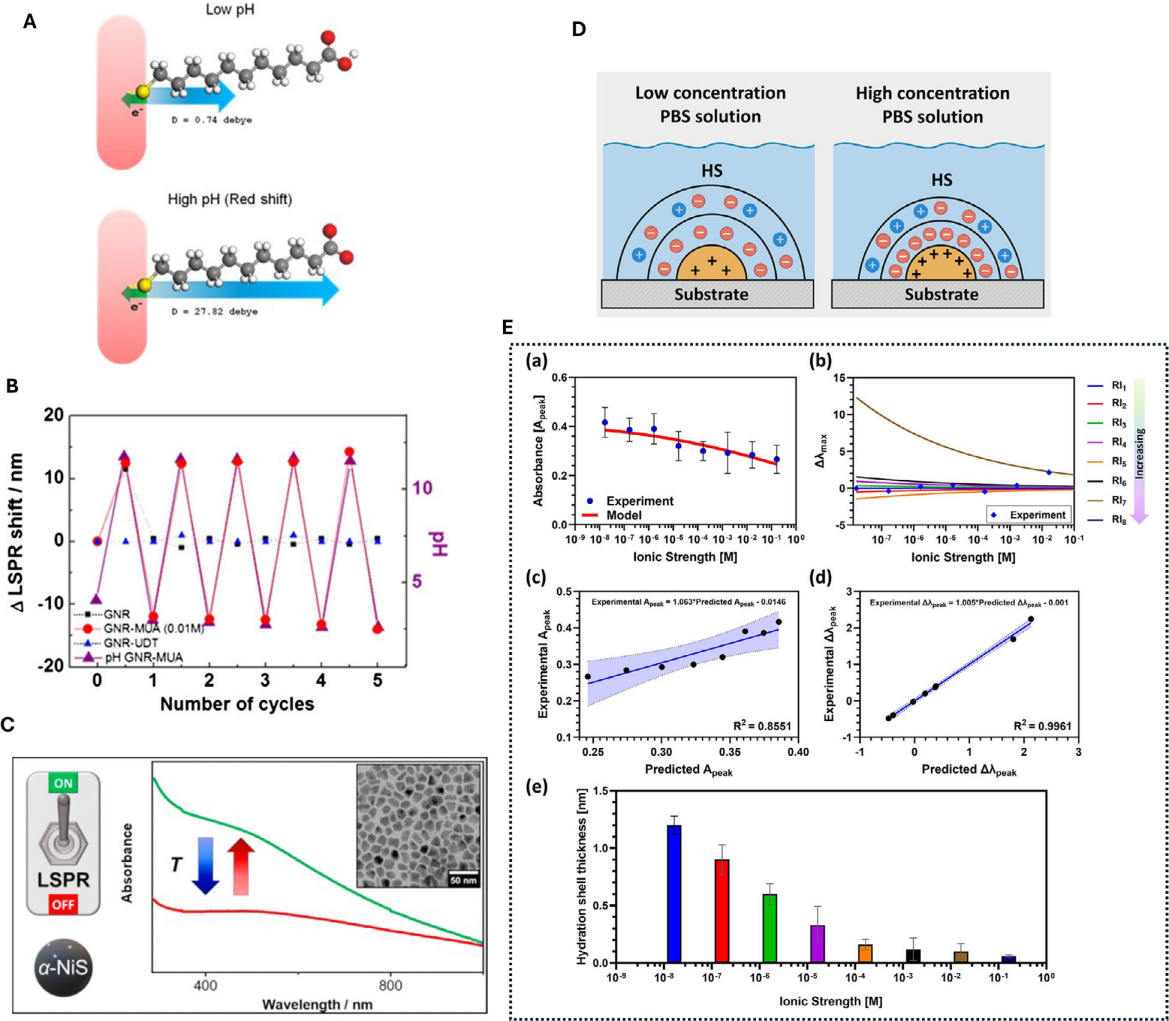
LSPR sensitivity to environment (A) Schematic illustrating the electron‐withdrawing force on GNR‐MUA, leading to a blue or redshift of the LSPR at low and high pH, respectively. (B) Reversibility of LSPR shift from GNP, GNP‐UDT, and GNP‐MUA between pH 2.60 and 11.75. A and B are reproduced under the terms of the CC‐BY license [45]. Copyright 2013, Nature Springer). (C) Schematic showing temeprature sensitivity of the LSPR nanstructures. Reproduced under the terms of the CC‐BY‐NC‐ND 4.0 [46]. Copyright 2021, ACS. (D) Schematic shows the formation of hydration layer on the LSPR nanostructures (dependent on ion concentration of the buffer) which can affect the sensitivity of sensor. (E)Modeling of (a) absorbance peak and (b) wavelength shift with increasing ionic strength. Linear regression analysis comparing experimental and predicted values of (c) absorbance peak and (d) wavelength shift. The solid line represents the best‐fit regression line, and the shaded area indicates the 95% confidence interval. (e) HS thickness variation with ionic strength. (D) and (E) are reproduced under the terms of the CC‐BY license [47]. Copyright 2025, Wiley.

Localized surface plasmon resonance sensors are not only responsive to specific molecular binding but also to any perturbation that modifies either the dielectric environment of the nanostructure or the intrinsic dielectric function of the metal. While this broadband sensitivity underlies the technique's utility, it also makes the signal vulnerable to environmental fluctuations unrelated to the biochemical interaction of interest. Among the most pervasive are changes in temperature, pH, and salinity, each of which can induce spectral shifts of comparable magnitude to those from low‐nanomolar binding events [43, 44], see Figure 7A–E. Distinguishing these cross‐sensitivities from true analyte binding signals is therefore essential for quantitative LSPR biosensing. For quantitative context, reported binding‐induced LSPR peak shifts in typical bioassays are frequently in the sub‐nm to few‐nm range, whereas temperature drift and buffer‐to‐buffer refractive‐index mismatch can also produce apparent shifts on the order of ∼0.1–several nm over practical assay timescales. Therefore, drift often sets the effective LoD unless it is actively controlled or corrected.

### 5.1 Temperature Effects

Temperature affects LSPR in two principal ways. First, it alters the refractive index of the surrounding medium, with a coefficient

(22) $$\alpha_{m}=\frac{\partial n_{m}}{\partial T}$$

typically on the order of $-10^{-4}$ RIU/K for aqueous buffers [48, 49]. Second, it modifies the dielectric function of the metal through changes in the conduction electron scattering rate [50]. In the Drude model,

(23) $$\varepsilon_{p}\left(\omega ,T\right)=\varepsilon_{\infty }-\frac{\omega_{p}^{2}}{\omega^{2}+i\gamma \left(T\right)\omega }$$

where the damping constant γ(T) increases approximately linearly with temperature due to enhanced electron‐phonon scattering:

(24) $$\gamma \left(T\right)\approx \gamma_{0}+\beta \left(T-T_{0}\right)$$

with β a material‐dependent constant. The total wavelength shift from a temperature change ΔT can thus be expressed as

(25) $$\Delta \lambda_{T}\approx S_{\mathrm{surf}}\;\alpha_{m}\;\Delta T+\left(\frac{\partial \lambda_{\mathrm{res}}}{\partial \gamma }\right)\beta \;\Delta T$$

where the first term captures the bulk refractive index contribution and the second term captures the intrinsic metal response. Depending on the sign and magnitude of $\alpha_{m}$ and $\partial \lambda_{\mathrm{res}}/\partial \gamma$, these contributions can partially cancel or reinforce, leading to either red‐ or blueshifts, see Figure 7C.

### 5.2 pH Effects

pH changes can influence LSPR signals indirectly through their effect on the refractive index of the buffer and directly through modification of the surface chemistry [51, 52]. For example, protonation or deprotonation of functional groups in the capture layer can alter the effective layer thickness and density, producing a measurable shift, see Figure 7A–B. The effective refractive index change from pH variation can be modeled as

(26) $$\Delta n_{\mathrm{eff},\mathrm{pH}}=\frac{\partial n_{\mathrm{layer}}}{\partial \mathrm{pH}}\;\Delta \mathrm{pH}\cdot \left[1-e^{-d/\ell_{d}}\right]$$

where $n_{\mathrm{layer}}$ is the refractive index of the capture layer.

In buffers with weak ionic strength, pH changes can also modulate the electrical double layer at the metal surface [53], altering the local ion concentration and, consequently, the local refractive index. This electrochemical contribution can be modeled using the Gouy–Chapman–Stern framework, where the surface potential $\psi_{0}$ depends on pH via the Nernst relation [54]:

(27) $$\psi_{0}\approx \psi_{\mathrm{ref}}-\frac{2.303k_{B}T}{e}\left(\mathrm{pH}-{\mathrm{pH}}_{\mathrm{ref}}\right)$$

Changes in $\psi_{0}$ modify the ion density profile ρ(z), which in turn perturbs Δn(z) in Equation (3).

### 5.3 Salinity Effects

Variations in salinity more generally, ionic strength, affect LSPR primarily through their impact on the refractive index of the medium and the thickness of the electrical double layer [55, 56, 57]. The bulk refractive index change from an ionic concentration change $\Delta C_{\mathrm{ion}}$ can be approximated by

(28) $$\Delta n_{\mathrm{bulk},\mathrm{ion}}\approx k_{\mathrm{ion}}\;\Delta C_{\mathrm{ion}}$$

where $k_{\mathrm{ion}}$ is an empirical coefficient dependent on the specific salt. In addition, the Debye length $\lambda_{D}$, which sets the characteristic scale of the double layer, is given by

(29) $$\lambda_{D}=\sqrt{\frac{\varepsilon_{r}\varepsilon_{0}k_{B}T}{2N_{A}e^{2}I}}$$

where I is the ionic strength [47]. An increase in salinity reduces $\lambda_{D}$, compressing the double layer and potentially altering the effective thickness d in Equation (4). This can change Δλ even if the bulk refractive index remains constant [58], see also Figure 7D,E.

### 5.4 Combined Environmental Influence

In realistic assays, temperature, pH, and salinity can vary simultaneously and interact nonlinearly [43, 59, 60]. The total environmentally induced shift can be modeled as

(30) $$\Delta \lambda_{\mathrm{env}}\approx \Delta \lambda_{T}+\Delta \lambda_{\mathrm{pH}}+\Delta \lambda_{\mathrm{ion}}+O\left(\Delta T\cdot \Delta \mathrm{pH},\text{…}\right)$$

where the cross terms capture coupling between parameters, such as temperature‐induced changes in buffer pH or ionic mobility. Please note that here O(ΔT·ΔpH,⋯) denotes higher‐order coupling (cross‐sensitivity) terms that arise when multiple environmental parameters vary simultaneously. One approach to mitigate environmental cross‐sensitivities is to use a reference channel experiencing the same environmental variations but without analyte binding, as described earlier. However, for parameters like temperature that also affect the metal's intrinsic dielectric function, the sensitivity of the reference and active channels may differ slightly, leaving residual artefacts. A more robust method is to integrate environmental sensors directly on‐chip: a thermistor or resistance temperature detector (RTD) for temperature [61], a miniaturized pH electrode or ISFET for pH [62], and a conductivity sensor for salinity [63]. The LSPR shift can then be corrected in real time:

(31) $$\Delta \lambda_{\mathrm{corr}}\left(t\right)=\Delta \lambda_{\mathrm{meas}}\left(t\right)-\left[k_{T}\Delta T\left(t\right)+k_{\mathrm{pH}}\Delta \mathrm{pH}\left(t\right)+k_{\mathrm{ion}}\Delta C_{\mathrm{ion}}\left(t\right)\right]$$

where the coefficients $k_{T}$, $k_{\mathrm{pH}}$, and $k_{\mathrm{ion}}$ (respective sensitivity co‐efficients) are determined from independent calibrations. The challenge and the reason this remains an unresolved conundrum is that the environmental coefficients are not strictly constant but can depend on the current state of the sensor surface, the bound analyte layer, and even the assay progress. For example, adsorption of proteins can change the thermal expansion coefficient of the local environment or alter the pH sensitivity by modifying the surface charge. As a result, a correction factor determined at the beginning of the assay may not remain valid throughout, leading to residual errors in the extracted binding curves. Addressing this issue will likely require a hybrid approach combining physical referencing, direct environmental monitoring, and adaptive signal processing algorithms that update the correction coefficients in real time. Such adaptive models could be based on recursive parameter estimation or machine learning frameworks that detect and compensate for slow drifts in sensitivity. Until such techniques are standardized and validated across laboratories, environmental cross‐sensitivities will continue to limit the quantitative reliability of LSPR biosensing, particularly in field‐deployed or point‐of‐care settings where environmental control is less stringent than in the laboratory. The dominant environmental cross‐sensitivities that limit LSPR accuracy, together with pragmatic mitigation and correction strategies, are summarized in Table 3.

**Table 3:** Comparison of key environmental cross‐sensitivities in LSPR biosensing and practical mitigation strategies.

Factor	Dominant mechanism(s)	Typical manifestation in LSPR readout	Practical mitigation / correction
Temperature	Thermo‐optic changes in the surrounding medium; temperature‐dependent metal dielectric function; local heating under illumination; viscosity‐driven transport changes	Baseline drift; apparent peak shifts during warm‐up or flow changes; run‐to‐run offsets if thermal history differs	Thermal control (PID stage, enclosure); allow equilibration; co‐measure temperature and compensate; use dual‐channel reference with shared thermal environment; minimise self‐heating (power control)
pH	Protonation/deprotonation alters surface charge and receptor conformation; modifies hydration and interfacial refractive index; affects binding kinetics and nonspecific adsorption	Baseline shifts correlated with buffer pH; changes in kinetic shape (association/dissociation); increased nonspecific adsorption	Strong buffering near operating pH; report and control pH at sensor inlet; use pH‐tolerant surface chemistries; include pH monitoring and correction; include negative‐control surfaces
Ionic strength / salinity	Electrostatic screening (Debye length changes) alters interaction range and adsorption; modifies double‐layer structure and hydration; affects colloidal stability and nonspecific adsorption	Apparent shifts during buffer exchange; altered affinity and kinetics; baseline offsets due to bulk refractive index and interfacial structure changes	Maintain constant ionic strength where possible; match running and sample buffers; conductivity monitoring; stepwise buffer exchange; reference channel and multiparametric correction; explicitly report salinity or ionic strength

#### 5.4.1 Examples of Multi‐Parametric Correction Schemes

Beyond single reference‐channel subtraction, multi‐parametric correction can be implemented in several practical ways, including data‐driven full‐spectrum regression and dual‐channel architectures that explicitly decouple temperature from the analyte‐sensitive channel [64, 65, 66, 67]. In practice, these approaches are complementary and can be combined depending on whether the platform provides single‐wavelength tracking, full spectra, and/or auxiliary environmental readouts.

*(i) Sensor‐fusion correction using co‐measured environmental variables*. A straightforward strategy is to record temperature, pH, and ionic strength (often proxied by conductivity) in parallel with the LSPR measurement, and then use these traces to correct baseline contributions caused by thermo‐optic drift, buffer mismatch, and electrostatic screening transients. This workflow is particularly helpful when injection steps introduce simultaneous bulk refractive‐index changes and binding, and when long assay durations amplify slow drift. Dual‐parameter designs that explicitly monitor temperature alongside the plasmonic signal provide an experimentally grounded pathway to such correction [66, 67].

*(ii) Dual‐channel/dual‐mode architectures to decouple confounders*. A hardware‐level route is to include a second optical channel designed to be minimally affected by binding while remaining sensitive to the dominant confounder (most commonly temperature). In fiber and integrated implementations, this can be realized by pairing an analyte‐sensitive LSPR response with an additional interferometric or geometry‐dependent response that primarily tracks temperature. The key practical advantage is that both channels share the same flow history and thermal environment, enabling more faithful compensation than remote referencing [66, 67].

*(iii) Full‐spectrum/multi‐metric correction (feature‐vector rather than peak shift)*. When full spectra are available, environmental perturbations and binding events can be separated more robustly by using multiple spectral descriptors rather than peak position alone. Bulk refractive‐index and temperature changes often appear as comparatively uniform spectral translations, whereas surface chemistry changes, fouling, or aggregation can also modulate linewidth and extinction amplitude through damping, scattering, or heterogeneous broadening. Using a multi‐metric representation (e.g., peak position together with linewidth and amplitude, centroid‐based metrics, or constrained spectral fits) enables regression‐ or learning‐based correction that is less sensitive to spectral congestion and peak‐picking artefacts [64, 65].

*(iv) Adaptive correction for time‐varying cross‐sensitivities*. Cross‐sensitivities are not always constant: surface conditioning layers (e.g., gradual protein adsorption), fouling, or changes in local charge screening can alter the apparent temperature and ionic‐strength responses over time. In such cases, correction can be improved by updating calibration factors during the assay using baseline windows, periodic buffer references, or slow‐drift models. This reduces residual curvature in long recordings and improves transferability between runs when the surface state differs.

Taken together, these examples emphasize that multi‐parametric correction is best viewed as a toolkit. The optimal choice depends on the available observables (single‐point tracking vs. full spectra), whether auxiliary environmental sensors can be integrated, and the assay regime (short injections vs. long kinetics), but in all cases the goal is the same: to reduce environmental degrees of freedom so that the remaining signal can be interpreted more quantitatively as a binding and surface‐process‐dominant response.

## 6 Reproducibility and Batch‐to‐Batch Variation

The spectral position, lineshape, and sensitivity of an LSPR sensor are acutely dependent on the precise size, shape, composition, and dielectric environment of the nanostructures used [68, 69, 70]. Even small deviations in these parameters between fabrication runs can result in measurable differences in the baseline resonance wavelength $\lambda_{0}$, the bulk refractive index sensitivity S, and the decay length $\ell_{d}$ [71]. Consequently, batch‐to‐batch variation in nanoparticle synthesis or lithographic fabrication can generate performance disparities large enough to obscure or confound biosensing signals [72]. This sensitivity to fabrication variance is one of the primary factors limiting the reproducibility and comparability of LSPR measurements across laboratories and even within a single facility over time.

**Figure 8:**
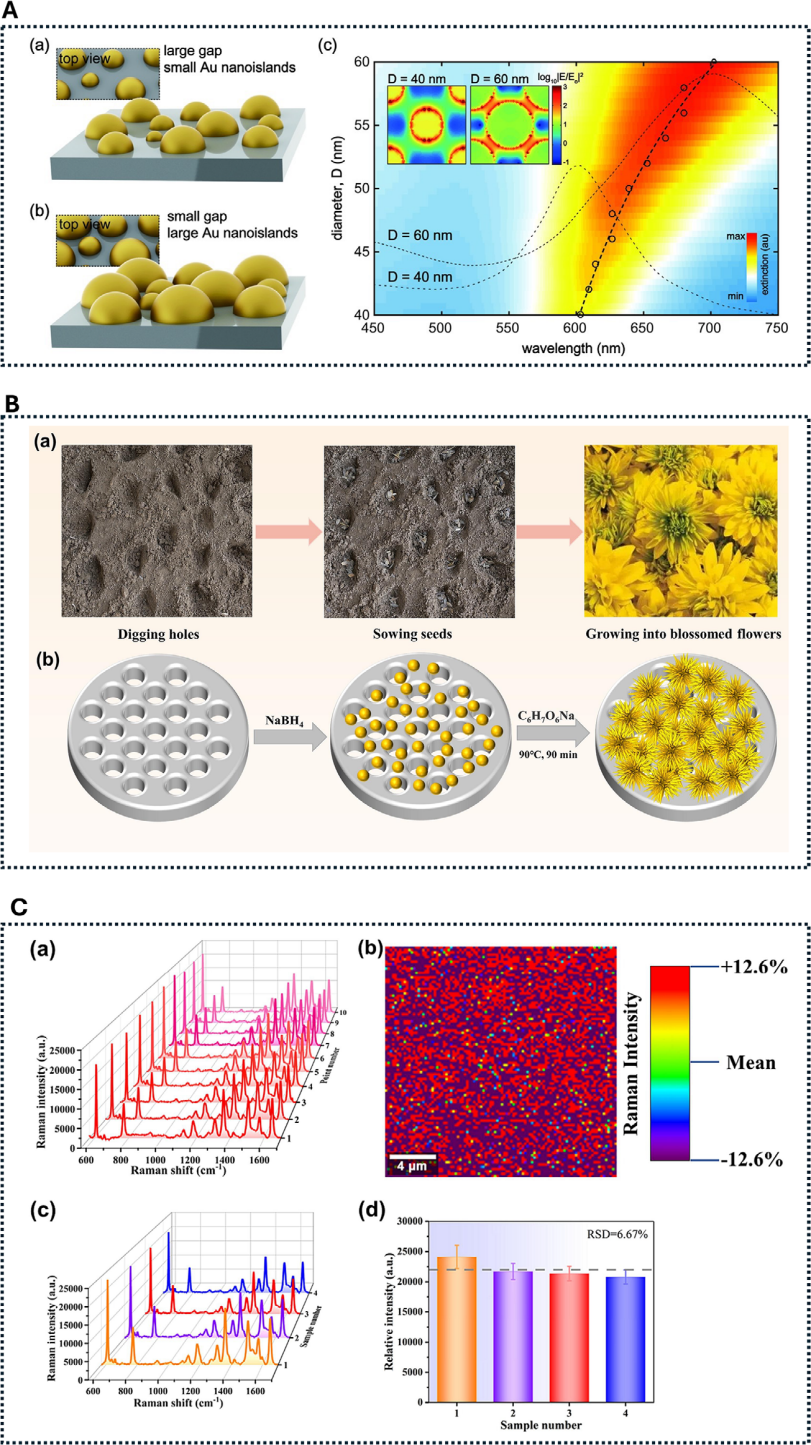
Morphological control and reproducibility in plasmonic nanostructures. (A) Schematic illustration of Au nanoislands formed via (a) single‐step solid‐state dewetting and (b) repeated dewetting. The repeated dewetting process produces larger islands with smaller interparticle gaps, increasing packing density. (c) Extinction spectra and plasmon resonance shifts as a function of nanoisland diameter, where the dashed curve shows the resonance wavelength shift and representative spectra for D=40 nm and D=60 nm. Insets display electric field intensity distributions at the plasmon resonance wavelength for D=40 nm and D=60 nm, respectively. Reproduced under terms of CC‐BY 4.0 [73]. Copyright 2015, Springer Nature. (B) (a) Schematic of the horticulturist's flower‐growing process, and (b) analogous scheme for preparing Au nanoflower (AuNF) substrates via ion track‐etched membrane‐assisted, seed‐mediated growth. (C) (a) Raman spectra of R6G collected at 10 random points on a single substrate. (b) Raman mapping of R6G for the peak at 612 ${\mathrm{cm}}^{-1}$ over a 20×20μm 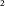 area. (c) Average Raman spectra of R6G acquired from four independently fabricated substrates. (d) Corresponding Raman intensities for the 612 ${\mathrm{cm}}^{-1}$ peak, where the horizontal dashed line indicates the mean value. The R6G concentration is $10^{-6}$ M. (B) and (C)Reproduced with permission from [74]. Copyright 2024, Elsevier

Figure 8 illustrates how fabrication protocols and morphological control directly affect the reproducibility of plasmonic nanostructures. In panel A, repeated solid‐state dewetting yields larger, more densely packed Au nanoislands, which in turn shift the LSPR peak due to changes in interparticle coupling and aspect ratio [73]. Panels B and C highlight how controlled growth and substrate preparation influence reproducibility: the Au nanoflower substrates produced via membrane‐assisted, seed‐mediated growth show high Raman uniformity across multiple points and batches [74]. Together, these examples demonstrate how both top‐down and bottom‐up fabrication methods can tune morphology but also introduce systematic variations that impact spectral response and sensing repeatability. The dependence of $\lambda_{0}$ on nanoparticle geometry can be described, in the quasistatic approximation, by the condition

(32) Reεp(ωres)=−1−LLεm

where $\varepsilon_{p}$ is the particle dielectric function, $\varepsilon_{m}$ is the dielectric constant of the medium, and L is the depolarization factor, which depends on the particle aspect ratio. For a sphere, L=1/3; for an ellipsoid, L varies with axis lengths a, b, and c. A change in aspect ratio ΔAR from batch‐to‐batch modifies L and thus $\lambda_{0}$. For small changes in L, the wavelength shift is approximately

(33) $$\Delta \lambda_{0}\approx \left(\frac{\partial \lambda_{0}}{\partial L}\right)\Delta L$$

with $\partial \lambda_{0}/\partial L$ determined by the slope of the dispersion relation. Because L is a nonlinear function of geometry, small deviations in nanofabrication parameters can translate into disproportionately large $\Delta \lambda_{0}$. Material composition also plays a critical role. In the Drude Lorentz model [75], the plasma frequency $\omega_{p}$ depends on the free electron density $n_{e}$:

(34) $$\omega_{p}=\sqrt{\frac{n_{e}e^{2}}{\varepsilon_{0}m_{e}^{*}}}$$

where $m_{e}^{*}$ is the effective electron mass. Unintentional alloying or contamination can change $n_{e}$, thereby shifting $\omega_{p}$ and $\lambda_{0}$. The relative change in resonance wavelength from a change $\Delta n_{e}$ is

(35) $$\frac{\Delta \lambda_{0}}{\lambda_{0}}\approx -\frac{1}{2}\frac{\Delta n_{e}}{n_{e}}$$

Even sub‐percent changes in $n_{e}$ can produce shifts of several nanometres, which is significant in the context of biosensing where binding signals are often of similar magnitude. Another parameter is the substrate supporting the nanostructures which can influence the local dielectric environment [76]. Variations in substrate refractive index $\varepsilon_{s}$ or the thickness of adhesion and functionalization layers can cause baseline shifts [77]. If the effective medium approximation is used to model the substrate‐medium interface, the resonance wavelength shift due to a change $\Delta \varepsilon_{s}$ is

(36) $$\Delta \lambda_{0}\approx S_{\mathrm{sub}}\;\Delta n_{s}$$

where $S_{\mathrm{sub}}$ is the substrate sensitivity coefficient and $\Delta n_{s}$ is the change in substrate refractive index. Differences in cleaning protocols, surface roughness, or oxide layer growth between fabrication batches can thus produce measurable spectral differences. Batch‐to‐batch variations in geometry and environment not only shift $\lambda_{0}$ but can also alter S and $\ell_{d}$ [78]. The bulk sensitivity S scales approximately as

(37) S∝λ0∂Re[εp]∂nm

so a change in $\lambda_{0}$ or in the slope of the dielectric function dispersion with respect to the medium index $n_{m}$ can alter S. Similarly, $\ell_{d}$ depends on field confinement, which is geometry‐dependent as in Equation (32). A change in aspect ratio or tip curvature between batches can thus change the effective sensing volume. From a reproducibility standpoint, batch‐to‐batch and site‐to‐site spreads in resonance position and linewidth are often non‐negligible. Laboratories commonly observe distributions in $\lambda_{0}$ and FWHM across nominally identical regions/particles, which propagate into variability in sensitivity and figures of merit and can shift the apparent response to identical binding events. To make such variability comparable across studies, we recommend reporting basic summary statistics (mean, SD and/or coefficient of variation) across n independent structures/regions (e.g., n≥10–30), together with the fitting method, instrument settings, and assay conditions.

### 6.1 Statistical Representation of Batch Variation

If $\lambda_{0}$, S, and $\ell_{d}$ vary stochastically from batch‐to‐batch, the measurement of a binding‐induced shift $\Delta \lambda_{\mathrm{surf}}$ will have an associated uncertainty due to fabrication:

(38) $$\sigma_{\mathrm{fab}}^{2}=\sigma_{\lambda_{0}}^{2}+{\left(\Delta n_{\mathrm{eff}}\right)}^{2}\sigma_{S}^{2}+{\left(S\Delta n_{\mathrm{eff}}\frac{d}{\ell_{d}^{2}}e^{-d/\ell_{d}}\right)}^{2}\sigma_{\ell_{d}}^{2}$$

where $\sigma_{\lambda_{0}}$, $\sigma_{S}$, and $\sigma_{\ell_{d}}$ are the standard deviations of $\lambda_{0}$, S, and $\ell_{d}$ across batches. This uncertainty adds in quadrature to other noise sources and can dominate the total error budget if fabrication processes are not tightly controlled. Mitigation strategies for batch variation fall into two categories: fabrication control and post‐fabrication calibration [79]. In the first category, advances in top‐down fabrication methods [80] such as electron‐beam lithography and nanoimprint lithography offer higher reproducibility in geometry but at higher cost and lower throughput compared to colloidal synthesis. For bottom‐up methods [81], improved seed‐mediated growth protocols, tighter temperature control, and automated reagent delivery systems can reduce geometric variability. In the second category, post‐fabrication calibration involves measuring each batch's $\lambda_{0}$, S, and $\ell_{d}$ before use in sensing experiments, then normalising the biosensing signals to these parameters. For example, if the expected shift for a given assay is proportional to S, then a normalized shift

(39) $$\Delta \lambda_{\mathrm{norm}}=\frac{\Delta \lambda_{\mathrm{meas}}}{S_{\mathrm{batch}}}$$

can remove first‐order sensitivity differences between batches. Similarly, baseline offsets in $\lambda_{0}$ can be removed by referencing each batch's initial spectrum to a standard wavelength scale. Despite these mitigation strategies, batch‐to‐batch variation remains a stubborn problem because not all relevant parameters are easily measurable or controllable. Nanoparticle tip curvature, crystallographic facet distribution, and surface roughness can influence LSPR in ways that are difficult to quantify post‐fabrication [82]. Furthermore, biosensing signals often depend on the details of the local surface chemistry, which can vary between batches even if the underlying nanostructure is identical [83]. Differences in ligand packing density, orientation, and stability can all alter the effective $\Delta n_{\mathrm{surf}}$ for a given binding event [84]. The conundrum, therefore, is that even with precise fabrication and calibration, residual differences between batches can still be large enough to limit the comparability of results, especially when signals are small. Until there is a universally adopted standard for nanoparticle characterization and calibration, including certified reference materials and agreed‐upon measurement protocols, the reproducibility of LSPR biosensing will remain constrained by fabrication variability.

#### 6.1.1 Toward Inter‐Laboratory Standardization: Practical Calibration Standards

To improve comparability across laboratories, a pragmatic approach is to adopt a small set of minimum calibration and reporting standards that remain valid regardless of the fabrication route (colloidal or lithographic) and the optical readout modality (extinction, scattering, or phase). I suggest three elements that cover most of the variability that otherwise makes results hard to compare: first, routine verification of the wavelength axis and instrument performance through regular spectrometer wavelength calibration, alongside clear documentation of spectral resolution, integration time, and the fitting method used, since nanometre‐scale offsets can be on the same order as many biosensing signals. Second, a bulk refractive‐index sensitivity standard, obtained by measuring $S_{\mathrm{bulk}}$ using traceable refractive‐index standards or well‐characterized refractive‐index liquids over the assay‐relevant range, while explicitly reporting temperature and buffer composition. Third, the use of reference nanostructures or reference materials, achieved by including benchmark plasmonic samples with well‐characterized size, shape, and optical response (for example, a reference nanoparticle suspension).

## 7 Quantification in Multiplexed or Crowded Spectra

**Figure 9:**
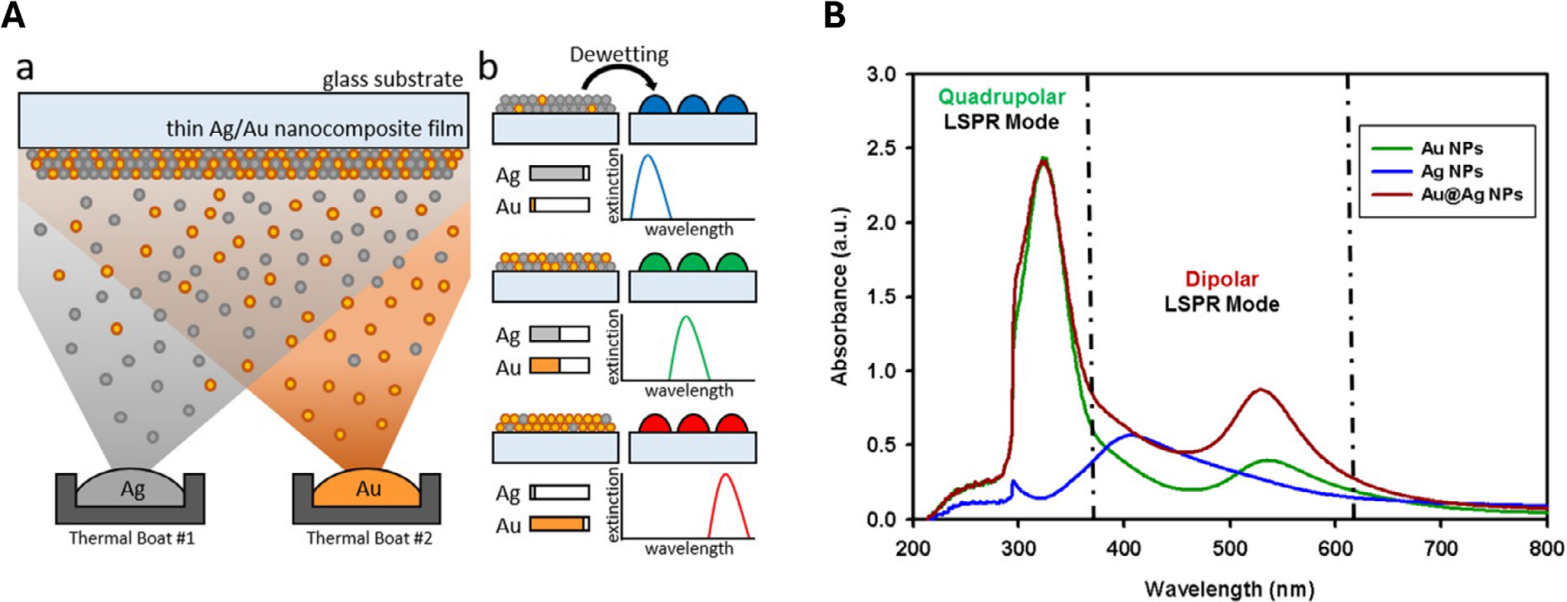
Spectral engineering and mode design in Multiplexed LSPR biosensing. (A)Formation of Ag–Au bimetallic nanostructures via thermal dewetting, enabling control of plasmonic mode composition. Reproduced reproduced with permission CC‐BY [86]. Copyright 2019, Springer Nature. (B) Extinction spectra of Au, Ag, and Au@Ag nanoparticles showing distinct quadrupolar and dipolar LSPR modes whose spectral overlap illustrates the onset of crowded spectra in multiplexed sensing. Reproduced reproduced with permission CC‐BY [87]. Copyright 2019, ACS.

One of the most attractive features of LSPR biosensing is the ability to design arrays of nanostructures with distinct resonance wavelengths, enabling multiplexed detection of multiple analytes within a single sample [19]. One can vary the geometry, composition, and arrangement of nanoparticles to engineer a set of plasmonic modes whose peak positions $\lambda_{0,i}$ are spectrally separated [85], see for example Figure 9A where composition of bimetallic nanostrutcures control the plasmonic mode composition. However, as the number of modes increases, their spectral features inevitably broaden and begin to overlap, leading to what is known as a crowded spectrum, Figure 9B. In such conditions, the quantitative extraction of individual peak shifts $\Delta \lambda_{i}$ becomes ill‐posed and small uncertainties in the fitting procedure can translate into large errors in the inferred binding signal, and cross‐talk between channels can produce apparent shifts in one mode when only another mode is physically perturbed.

To understand the spectral overall and peak broadening we first need to model the multiplexed LSPR assay. The extinction or scattering spectrum of a multiplexed array can be modeled as the sum of Lorentzian (or Fano‐type) lineshapes for each mode  [34, 88]:

(40) $$I\left(\lambda \right)=\sum_{i=1}^{N}\frac{A_{i}}{1+{\left[\frac{\lambda -\lambda_{0,i}}{\Gamma_{i}/2}\right]}^{2}}$$

where $A_{i}$ is the amplitude, $\lambda_{0,i}$ is the centre wavelength, and $\Gamma_{i}$ is the full width at half maximum (FWHM) of mode i. As the number of modes N increases or as $\Gamma_{i}$ becomes large relative to the spacing between $\lambda_{0,i}$, the spectral overlap increases. When two peaks are close together, a shift in one peak position $\Delta \lambda_{j}$ modifies the apparent position of its neighbor i≠j during fitting. The cross‐talk coefficient $C_{ij}$ can be defined as:

(41) $$C_{ij}=\frac{\partial \lambda_{0,i}^{\mathrm{fit}}}{\partial \lambda_{0,j}}$$

where $\lambda_{0,i}^{\mathrm{fit}}$ is the fitted peak position. In a perfectly orthogonal system, $C_{ij}=0$ for i≠j, but in crowded spectra $|C_{ij}|$ can be substantial. Therefore, extracting $\Delta \lambda_{i}$ from crowded spectra is typically done *via* nonlinear least‐squares fitting of Equation (40) to the measured I(λ). The covariance matrix Σ of the fitted parameters reveals the degree of ill‐posedness:

(42) $$\Sigma_{ij}\approx \sigma^{2}{\left[{\left(\frac{\partial I}{\partial p_{i}}\right)}^{T}\left(\frac{\partial I}{\partial p_{j}}\right)\right]}^{-1}$$

where $p_{i}$ denotes the fit parameters ($\lambda_{0,i}$, $\Gamma_{i}$, $A_{i}$) and σ is the noise level. Large off‐diagonal elements $\Sigma_{ij}$ indicate strong correlations between parameter estimates, meaning that noise in one mode's parameters will propagate into others. Given the challenges of deconvolving overlapping peaks, alternative spectral metrics have been proposed. One is the spectral centroid $\lambda_{\mathrm{centroid}}$ over a predefined wavelength band $\left[\lambda_{a},\lambda_{b}\right]$:

(43) $$\lambda_{\mathrm{centroid}}=\frac{\int_{\lambda_{a}}^{\lambda_{b}}\lambda \;I\left(\lambda \right)\;d\lambda }{\int_{\lambda_{a}}^{\lambda_{b}}I\left(\lambda \right)\;d\lambda }$$

While less sensitive to noise than individual peak positions, the centroid averages over contributions from all modes in the band and thus sacrifices selectivity. Another approach is curvature analysis, in which the second derivative of the spectrum is examined:

(44) $$\kappa \left(\lambda \right)=\frac{I^{\prime \prime }\left(\lambda \right)}{{\left[1+I^{\prime }{\left(\lambda \right)}^{2}\right]}^{3/2}}$$

where $I^{\prime }\left(\lambda \right)$ and $I^{\prime \prime }\left(\lambda \right)$ are the first and second derivatives [89]. Peaks and shoulders in κ(λ) can sometimes resolve modes more clearly than the raw spectrum. Recent works have also explored machine learning approaches [90, 91, 92, 93] to deconvolving crowded spectra. In a supervised learning framework, a model is trained on simulated or experimental spectra with known $\lambda_{0,i}$ values. The input spectrum I is mapped to the output vector of peak shifts Δλ:

(45) $$\Delta \lambda =f_{\mathrm{ML}}\left(I;\theta \right)$$

where $f_{\mathrm{ML}}$ is the machine learning model (e.g., a convolutional neural network) with parameters θ. Such models can, in principle, learn to disentangle overlapping contributions if trained on sufficiently diverse and realistic datasets. Even without machine learning, cross‐talk can be quantified and corrected by constructing the cross‐sensitivity matrix C whose elements are given by Equation (41). If the measured shifts $\Delta \lambda^{\mathrm{meas}}$ are related to the true shifts $\Delta \lambda^{\mathrm{true}}$ by

(46) $$\Delta \lambda^{\mathrm{meas}}=C\;\Delta \lambda^{\mathrm{true}}$$

then, provided C is invertible, one can estimate the true shifts via

(47) $$\Delta \lambda^{\mathrm{true}}\approx C^{-1}\;\Delta \lambda^{\mathrm{meas}}$$

However, in practice, C may be ill‐conditioned in crowded spectra, amplifying noise when inverted. It should also be noted that degree of spectral crowding is not solely determined by the number of modes but also by their linewidths and relative amplitudes. Narrower linewidths $\Gamma_{i}$ reduce overlap but may also reduce the signal‐to‐noise ratio if the mode's scattering cross‐section is low. The figure of merit for mode i in a multiplexed context can be defined as

(48) $${\mathrm{FOM}}_{i}=\frac{S_{i}}{\Gamma_{i}}$$

where $S_{i}$ is the sensitivity of mode i. Designing modes to maximize ${\mathrm{FOM}}_{i}$ while maintaining adequate separation from neighbors is a key engineering challenge. Even with optimal mode design, careful calibration, and advanced deconvolution algorithms, quantification in multiplexed LSPR remains fundamentally more challenging than in single‐mode systems. The conundrum lies in balancing the desire for high multiplexing capacity with the need for quantitative precision. As the number of modes N increases, the dimensionality of the fitting problem grows, and so does the potential for parameter correlation and error propagation. Without universally accepted metrics for cross‐talk tolerance and deconvolution reliability, results from multiplexed systems will remain difficult to compare across different platforms and studies. Progress in this area will likely come from an integrated approach combining electromagnetic simulation to design orthogonal mode sets, robust statistical methods to quantify uncertainty in crowded spectra, and standardised calibration protocols using reference samples with known refractive index perturbations. Until such practices are widely adopted, the interpretation of crowded LSPR spectra will remain as much an art as a science.

## 8 Conclusions and Outlook

The six conundrums discussed in this work highlight the fundamental challenge of translating the rich physics of localized surface plasmon resonance into reproducible and quantitative biosensing. Although each puzzle–from the ambiguity of red vs. blueshifts to the ill‐posed nature of multiplexed spectral deconvolution–has distinct mechanistic origins, they share a common feature: a single measured spectral observable often integrates multiple, competing physical effects. This coupling undermines the conventional assumption that an LSPR peak shift can be uniquely and directly mapped to a local refractive‐index change. Collectively, these challenges demonstrate that peak wavelength alone is frequently an insufficient descriptor of the LSPR response. Charge effects, near‐field confinement, morphological evolution, environmental perturbations, and finite decay‐length filtering can all contribute on comparable scales to the biochemical signal of interest. As a result, quantitative interpretation is limited not merely by experimental artefacts, but by intrinsic properties of plasmonic fields and their interaction with complex, dynamic environments.

Looking forward, these conundrums point to several clear research directions. First, there is a strong need for multidimensional observables that go beyond a single spectral metric. Parameters such as linewidth, amplitude, spectral curvature, polarization response, and angular dispersion provide complementary information that can help disentangle overlapping mechanisms when analyzed jointly. Second, engineered referencing strategies–such as paired plasmonic modes or self‐referencing architectures–offer a powerful route to suppress drift and environmental cross‐sensitivities by design rather than post‐hoc correction. Third, advances in data‐driven analysis, including multivariate statistics and machine‐learning models, hold promise for interpreting complex spectral responses, provided they are trained on physically informed and representative datasets. Standardization emerges as a critical enabler for progress. The field would benefit from certified plasmonic reference materials with well‐defined optical responses, standardized reporting of LSPR metrics beyond peak position, and benchmark datasets for evaluating spectral deconvolution and multiplexing algorithms. Such practices would facilitate meaningful comparison across platforms, fabrication routes, and laboratories. Ultimately, resolving these conundrums will require coordinated advances in nanofabrication, surface chemistry, optical instrumentation, and data analysis, embedded within a framework of rigorous standardization and inter‐laboratory validation. While the multi‐physics nature of LSPR sensing presents undeniable challenges, it also offers opportunities: by embracing this complexity rather than oversimplifying it, LSPR biosensing can evolve from a largely qualitative technique into a robust, quantitative, and universally comparable tool for label‐free biomolecular detection.

## Conflicts of Interest

The authors declare no conflicts of interest.
